# A “Wait-and-See” Approach to Quiescent Single-System Langerhans Cell Histiocytosis to Spare Children From Chemotherapy

**DOI:** 10.3389/fped.2020.00466

**Published:** 2020-08-12

**Authors:** Bernice Oh, Shawn Lee, Yuhe Ke, Miriam Kimpo, Allen Yeoh, Thuan Chong Quah

**Affiliations:** ^1^Viva-University Children's Cancer Centre, Khoo Teck Puat-National University Children's Medical Institute, National University Hospital, National University Health System, Singapore, Singapore; ^2^Department of Anesthesiology and Perioperative Medicine, Singapore General Hospital, Singapore, Singapore

**Keywords:** Langerhans cell histiocytosis, long-term outcome, skull neoplasms, spontaneous remission, children

## Abstract

**Background:** Langerhans Cell Histiocytosis (LCH) is a childhood disorder of histiocytes that is generally treated with systemic chemotherapy. Spontaneous resolution has been previously reported in Single System LCH (SS-LCH), which is less aggressive than multisystem disease. However, there are no clear guidelines on which patients can be safely spared from systemic chemotherapy. Here, we propose a risk stratification framework based on disease quiescence as determined by clinical and biochemical features of inflammation, to identify low risk patients who may be potentially spared from chemotherapy through a conservative “wait-and-see” approach.

**Methods:** Retrospective analysis in a single institution was conducted in children with SS-LCH, comparing features of inflammation and outcomes of those who received chemotherapy vs. those with quiescent disease, who were managed conservatively.

**Results:** Of 44 children with SS-LCH, only patients without risk-organ involvement were considered for conservative management. A “wait-and-see” approach was adopted for patients with quiescent disease as defined by clinical and biochemical evidence of disease activity. Following 2 weeks of watchful observation, decisions were made to either start treatment or continue conservative management. Based on data collected at diagnosis, patients with quiescent disease had a lower mean platelet count 339 × 10^9^/L (95%C.I: 285–393) vs. 482 × 10^9^/L (95% C.I: 420–544) *p* < 0.01, a lower mean white cell count 9.3 × 10^9^/L (95%C.I: 7.5–11.1) vs. 13.1 × 10^9^/L (95%C.I: 11–15.2) *p* < 0.01 and lower Erythrocyte-Sedimentation-Rate (ESR) 8.2 mm/h (95%C.I: 5.4–11) vs. 53.7 mm/h (95%C.I: 11–96.3) *p* = 0.04, suggesting that these are potential biochemical markers of disease activity. Other features of disease quiescence noted were rapid progression, functional disability, presence of a skull depression rather a lump and the lack of fever.

**Conclusions:** Further studies are required to validate our proposed framework to determine disease activity in SS-LCH. Within the limits of this current analysis, it appears that low-risk patients with clinically and biochemically quiescent SS-LCH, may potentially be spared from chemotherapy with good long-term outcomes.

## Introduction

Langerhans Cell Histiocytosis (LCH) in childhood can present with a wide spectrum of clinical manifestations with varying treatment outcomes that is largely dependent on disease severity. While fulminant multi-system involvement can be life threatening and progress very aggressively; single-system disease runs a more benign course and can spontaneously resolve.

Langerhans-cells were first described by Paul Langerhans in the 1868 (1) as epidermal cells—that we now know as dendritic cells—which have the ability to migrate from the epidermis to lymph nodes where they present antigens to T-cells. The key driving abnormality in LCH is the dysregulated clonal proliferation of LCH cells. Based on genetic studies of BRAF mutations in LCH patients, high-risk LCH (2) has been shown to arise from a mutated hematopoietic progenitor, whereas low-risk LCH arises from mutated tissue-specific early dendritic cells (3), suggesting that low-risk LCH has a different pathogenesis.

Observational studies of children with single-system-LCH (SS-LCH) report that some of these patients can be managed conservatively (4–7) but decisions as to who receives local or systemic treatment vs. no treatment within this subgroup is still largely based on physician discretion (8). Other reported cohort studies of SS-LCH have either only focused on groups of patients who received chemotherapy (9), patients with single bony “CNS-risk” lesions (10), or patients with only single-site bony lesions (11).

Current guidelines (12) recommend systemic chemotherapy for SS-LCH based on type and extent of organ involvement: “CNS-risk” sites, multifocal bone lesions and “special-site” lesions (functionally critical anatomical sites). However, earlier albeit limited, studies have also described elevated erythrocyte sedimentation rate and thrombocytosis to be possible indicators of disease activity in LCH (13). In order to further distinguish a subgroup of patients quiescent SS-LCH who may be spared from systemic chemotherapy, a modified approach based on clinical and biochemical features of disease activity in addition to current guidelines may be helpful.

Here, we report our institutional experience in managing pediatric patients with SS-LCH with and without systemic chemotherapy. In addition, we also propose a practical clinical decision-making framework to identify patients with quiescent SS-LCH without risk-organ involvement—who can be managed more conservatively with a “wait-and-see” approach.

## Methods and Definitions

We conducted a retrospective review of medical records of patients managed at the Department of Pediatrics at the National University Hospital in Singapore from 1985 to 2018. Patients were identified from physician records and diagnostic coding in electronic medical records. Patient data were extracted based on physician clinic lists and following review of scanned medical records and electronic records that were available at time of data extraction. This study was approved by the Domain Specific Review Board for research ethics in our institution.

LCH was diagnosed based on histological evidence of Langerhans-cells present in biopsy samples, immunohistochemical evidence of CD1a and S100 staining; or based on characteristic X-ray appearance of an eosinophilic granuloma for isolated skull vault lesions (14, 15). Patients without a histological diagnosis were only presumed to have LCH, if the characteristic lesions had resolved on follow-up imaging. The main differentials for isolated osteolytic lesions of the skull vault in children who were otherwise well, were that of fibrous dysplasia as well as bone cysts, which do not spontaneously resolve (16). All patients including those without a histological diagnosis received standard work up for LCH consisting of a full skeletal survey and other blood investigations also to exclude infection or other malignancy with osteolytic bony metastases.

Single-system LCH was defined as per current guidelines (12), when there was only one system involved at point of diagnosis (Bone, Skin, Lymph Nodes). Multisystem LCH was diagnosed if there were two or more organs or systems involved and were excluded from this analysis.

In our proposed criteria, fever was defined as an oral temperature of 38.0°C for at least 1 h and functional disability was defined as: (1) In older children: Inability to walk or perform activities of daily living (2) In babies: difficulties with feeding or age appropriate motor functions.

## Statistical Methods

Comparisons of the presenting features and indicators of disease activity at diagnosis between our patient groups were performed using student's *t*-tests. Statistical tests were two tailed and *p*-values that were <0.05 were considered statistically significant. Data analysis was performed using STATA SE 14.0.

## Results

Patients under the age of 18 who were diagnosed with LCH from the January 1985 to October 2018 were included in this data collection and analysis. 65 children were diagnosed with and/or treated for LCH at the Department of Pediatrics, National University Hospital in Singapore, of which 44 were diagnosed with single-system LCH.

Median age at diagnosis in the cohort was 3.0 years (range: 0.13–18 years). Toddlers aged 1–3 years formed the largest proportion in our cohort. There was a wide range of ethnicities within our study cohort as our institution receives referrals from around the South East Asia Region. Of the 44 patients who had single-system LCH, the bones were the most commonly affected system (86.4%, 38/44), out of which isolated lesions of the skull were commonest. In patients with multifocal bone lesions, the commonest sites of involvement were the long bones (45.4%, 5/11) and CNS-risk lesions (45.4%, 5/11).

**Figure 1:**
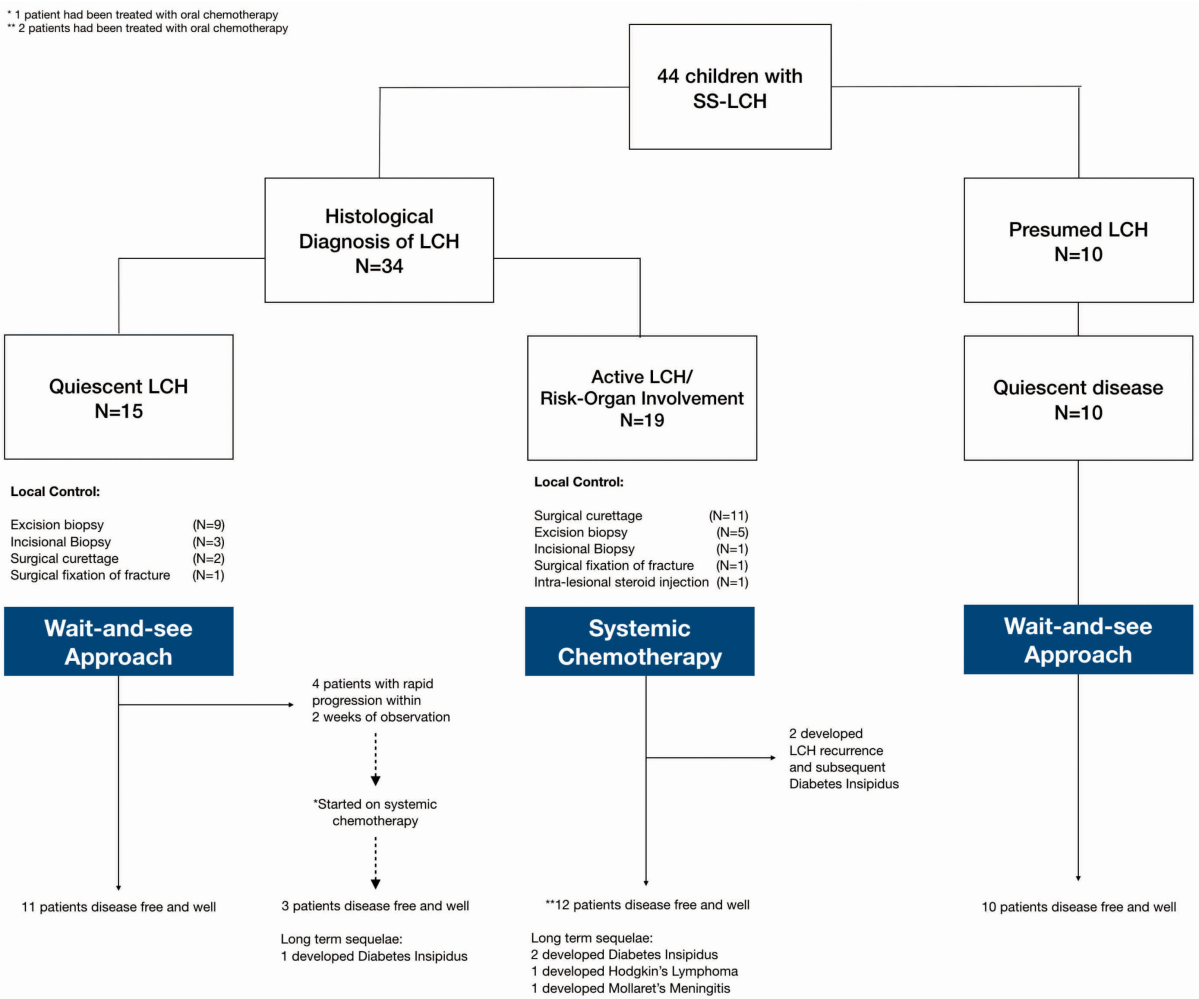
Description of patient groups, interventions and outcomes.

Our analysis focused on three main groups (Figure 1): (1) histologically confirmed LCH with quiescent disease, (2) histologically confirmed LCH with active disease or risk organ involvement requiring systemic treatment, and (3) those with presumed LCH. Baseline characteristics of patients in the three patient groups are described in Table 1.

**Table 1:** Indicators of disease quiescence in single-system LCH.

**Clinical indicators of disease quiescence**
• Lack of rapid progression within 2 weeks
• No severe pain or functional disability (feeding, age-appropriate motor functions, walking, activities of daily living)
• No fever^*^ (defined as temperature >38.0°C for at least 1 h)
• Presence of skull depression for skull vault lesions
Biochemical evidence of inflammation^*^
• Leukocytosis (total white count >20,000 × 10^9^/L)
• Thrombocytosis (platelet count >400 × 10^9^/L)
• Elevated erythrocyte sedimentation rate (ESR)/C-reactive protein (CRP)

* *In the absence of any other contributing factors, such as concomitant infections or any other underlying disease conditions that may cause elevation of inflammatory indicators*.

**Figure 2:**
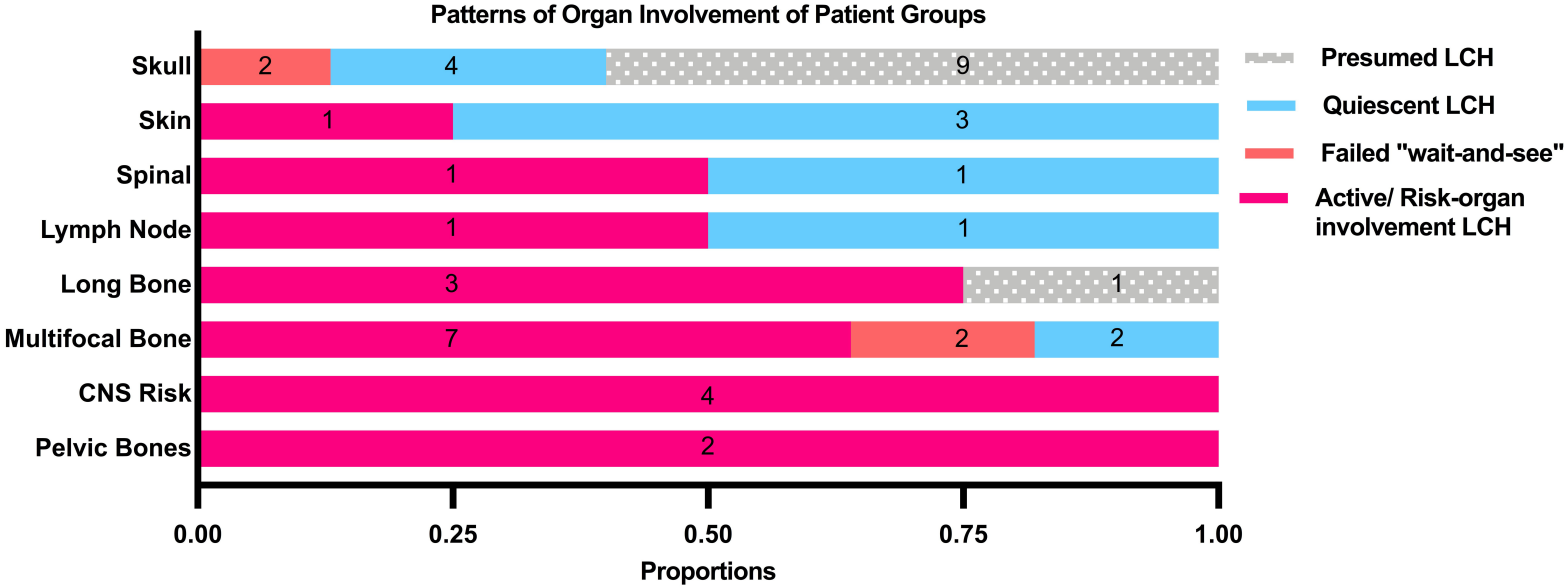
Patterns of organ involvement of the patient groups.

Patients who were diagnosed based on characteristic radiological features without a histological diagnosis were presumed to have LCH and were analyzed separately. These 10 patients did not have a histological diagnosis due to parental refusal of surgery for histological diagnosis (*n* = 8) and spontaneous resolution on repeat imaging from time of initial referral, before biopsy could be offered (*n* = 2). These patients with presumed LCH had an isolated clavicular lesion (*n* = 1) or isolated skull vault lesions (*n* = 9), of which seven presented with a skull depression (Table 2 and Figure 2). They were all clinically well with no other infective or constitutional symptoms to suggest an underlying osteomyelitis or malignancy with bony metastases. They remained on follow up till time of spontaneous resolution and were managed with a diagnosis of SS-LCH (as other osteolytic lesions, such as fibrous dysplasia and bone cysts do not spontaneously resolve). Of note, five of these nine isolated skull vault lesions were incidental findings on skull X-rays performed as part of evaluation for minor head injury.

**Table 2:** Baseline characteristics.

	**No chemotherapy** **(*N* = 15)**	**Systemic chemotherapy** **(*N* = 19)**	**Presumed LCH** **(*N* = 10)**
**Age (years)**	3.3	4.3	6.3
<1	20% (3/15)	16% (3/19)	10% (1/10)
1–10	80% (12/15)	74% (14/19)	60% (6/10)
>10	0	10% (2/19)	30% (3/10)
**Sex**			
Female	33% (5/15)	53% (10/19)	40% (4/10)
Male	67% (10/15)	47% (9/19)	60% (6/10)
**Ethnicity**			
Chinese	47% (7/15)	57% (11/19)	70% (7/10)
Vietnamese	0	21% (4/19)	0
Malay	20% (3/15)	11% (2/19)	0
Indian	7% (1/15)	0% (0/19)	10% (1/10)
Caucasian	13% (2/15)	0% (0/19)	20% (2/10)
Others	13% (2/15)	11% (2/19)	0
**Underlying/Past medical history**	7% (1/15)	17.4% (3/19)	0
**Significant family history**^**§**^	7% (1/15)	13.0% (2/19)	0
**Type of lesion**			
Isolated skull lesions	40% (6/15)^‡^	0	90% (9/10)^∧^
Skull depression	0	5% (1/19)	70% (7/10)
Isolated skin lesions	20% (3/15)	5% (1/19)	0
Isolated spinal lesions	7% (1/15)	5% (1/19)	0
Lymph node involvement	7% (1/15)	5% (1/19)	0
Isolated long bone lesions	0	16% (3/19)^**^	10% (1/10) (Clavicle)
Multifocal bone lesions	27% (4/15)	37% (7/19)	0
Isolated pelvic bone lesions	0	11% (2/19)	0
Isolated CNS-risk lesions	0	21% (4/19)	0
**Mean number of lesions**	1.9	2.2	1
**Mean widest diameter on X-ray (cm)**	2.9	4.0	2.2
**Mean duration of symptoms prior to presentation (weeks)**	4.4	2.7	4
Mean duration since diagnosis (years)	11.9 (1.6–19.9)	10.3 years (1.1–31.5)	6.3 (2–10.6)

§ *Patients with significant family history: One had an identical twin sibling with aggressive multi-system LCH, one had a sibling with a history of malignancy, and one was initially offered a watch and see approach but later received systemic therapy had a sibling with Kimura Disease*.

*Isolated Skull Lesions (No Systemic Treatment): Parietal bone (n = 3), Occipital bone (n = 2), Frontal bone (n = 1)*.

∧ *Isolated Skull Lesions (Presumed LCH): Occipital bone (n = 1), Parietal bone (n = 5), Temporal bone (n = 2), Frontal bone (n = 1)*.

** *Isolated Long Bone Lesions (Systemic Treatment): 1 patient had an isolated clavicle lesion, 2 patients had isolated femoral lesions*.

## A “Wait and See” Approach for Quiescent Single-System LCH

**Figure 3:**
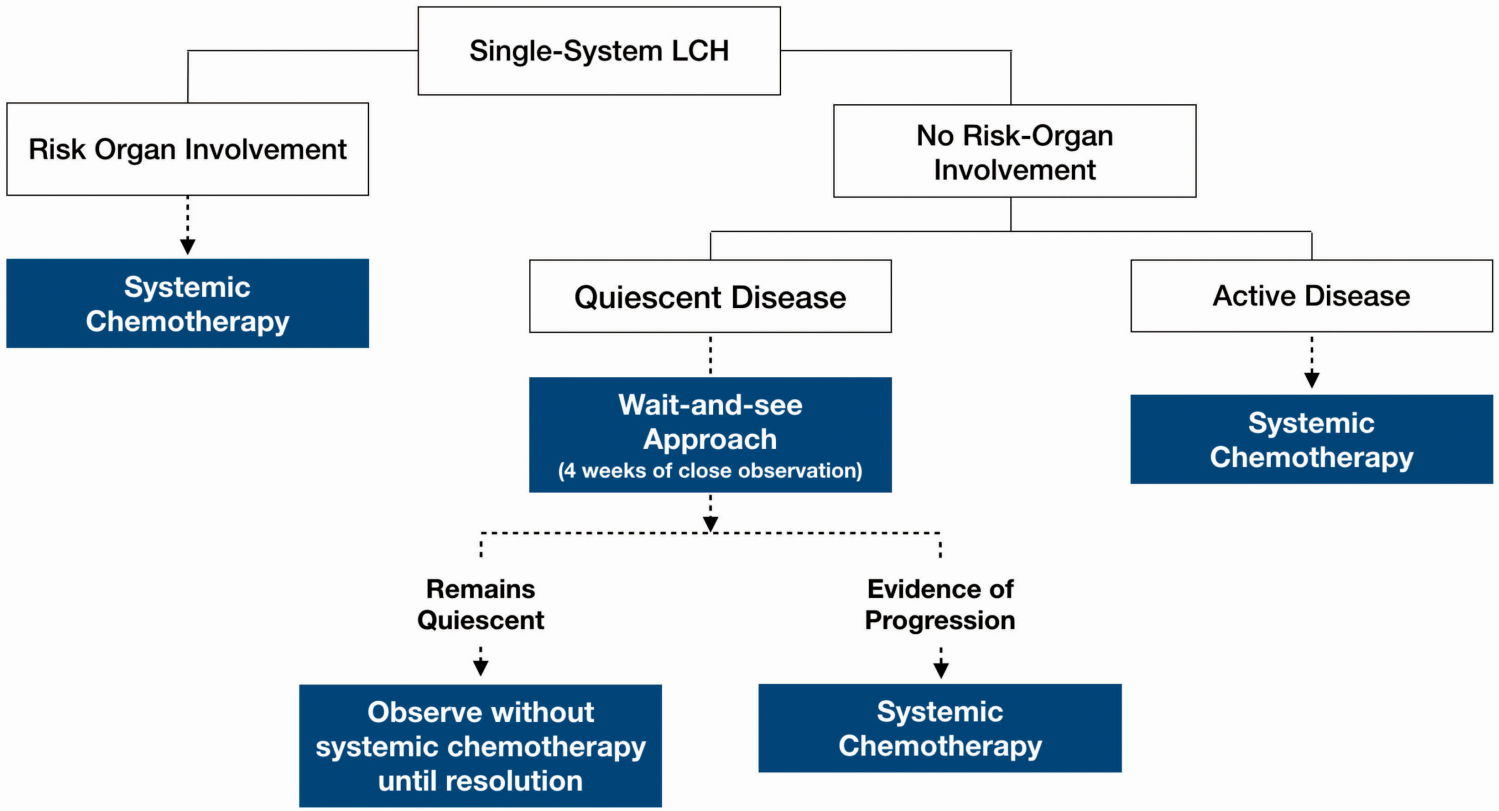
Proposed clinical decision-making framework.

47.7% of all patients in our cohort achieved spontaneous remission with a “wait-and-see” approach without need for any systemic chemotherapy, although almost half of these patients had presumed LCH without histological diagnosis and hence were not included in the analysis of indicators of disease quiescence. The majority of the patients who did not require treatment were those with isolated skull lesions (Figure 3).

These patients who did not require systemic chemotherapy were followed up for a mean total duration of 5.2 years (range 1–18 years). The mean frequency of follow up was every 3.1 months and parents were given return advice if there were any new or worsening symptoms. At every follow-up, patients underwent a complete clinical examination (specifically looking for the development of DI) and had a full blood count tested. They also had a 6–12 monthly skeletal survey.

Figure 1 Amongst these patients with quiescent disease, local control was as follows: (1) Topical steroids for skin lesions, (2) Surgical curettage for two patients (one had an isolated vertebral lesion and another had multifocal bony lesions and curettage at the biopsy site), (3) Surgical fixation of pathological fracture, (4) incisional/excisional biopsies. None received intra-lesional steroids.

Further analysis revealed several distinguishing features between the quiescent and active disease groups at diagnosis: (1) functional disability, (2) thrombocytosis, (3) leukocytosis, and (4) raised ESR (Table 3). There was also a larger proportion of patients in the active disease group who had features of pain, rapid progression in the 2 weeks preceding diagnosis and fever not attributed to other causes, although a significantly different. Based on these findings, patients with these features were more likely to have received systemic treatment for active disease.

**Table 3:** Analysis of indicators of disease quiescence.

	**Proportions/Mean** **±** **SD (95% C.I)**		***p*-values**
	**No chemotherapy (*N* = 15)**	**Systemic chemotherapy (*N* = 19)**	
**PRESENTING SYMPTOMS PRIOR TO HISTOLOGIC DIAGNOSIS**			
Rapid progression within 2 weeks	0.4 (6/15)	0.47 (9/19)	n.s
Functional disability	0	0.32 (6/19)	0.02
Fever (not attributed other causes)	0.07 (1/15)	0.21 (4/19)	n.s
Pain at primary site(s)	0.33 (5/15)	0.37 (7/19)	n.s
**BIOCHEMICAL EVIDENCE OF INFLAMMATION**			
Mean CRP (mg/L)	17.5 ± 8.5 (−90.5–125.5)	11 ± 7.85 (3.74–18.26)	n.s
Mean ESR (mm/h)	8.2 ± 2.28 (5.37–11.03)	53.67 ± 40.67 (10.99–96.34)	0.04
**FULL BLOOD COUNT AT DIAGNOSIS (× 10**^**9**^ **PER LITER)**			
Mean platelet count	339.07 ± 93.41 (285.14–393)	482.11 ± 124.1 (420.4–543.83)	0.001
Mean total white count	9.29 ± 3.06 (7.52–11.05)	13.07 ± 4.2 (10.98–15.16)	0.008
Mean neutrophil count	4.33 ± 2.24 (3.61–6.28)	6.17 ± 2.96 (4.7–7.64)	n.s
Mean lymphocyte count	4 ±1.92 (2.84–5.17)	5.2 ± 3.19 (3.61–6.79)	n.s
Mean eosinophil count	0.27 ± 0.19 (0.16–0.4)	0.21 ± 0.24 (0.09–0.34)	n.s
Mean basophil count	0.09 ± 0.1 (0.03–0.16)	0.06 ± 0.04 (0.04–0.08)	n.s
Mean monocyte count	0.66 ± 0.23 (0.51–0.82)	0.74 ± 0.34 (0.56–0.91)	n.s
Mean hemoglobin	11.94 ± 1.86 (10.87–13.02)	12.16 ± 1 (11.66–12.66)	n.s

*All 6 patients had lesions involving either the pelvic bones (n = 3) or femur (n = 3) that caused inability to weight bear or walk due to pain*.

Of the 15 patients with quiescent LCH at diagnosis, 4 (26.7%) patients aged 3.25–6 years old subsequently required systemic therapy in view of rapid progression of lesions during the “wait-and-see” period. Of these four patients, two had multifocal bony lesions in the skull and long bones without CNS risk organ involvement; and were initially thought to have quiescent disease in the absence of fever, thrombocytosis or raised inflammatory markers. The remaining two patients had isolated skull lesions which were also initially thought to be quiescent even though there was thrombocytosis, because they were asymptomatic and other inflammatory markers, such as ESR and CRP were not elevated. All four patients soon developed rapid and tender enlargement of one or more of their primary lesions within a mean duration of 1.25 weeks observation. They were then started on systemic treatment leading to durable long term LCH remission. One of the patients with multifocal bony lesions eventually developed DI 6 months after completing treatment, but remains otherwise well.

## Proposed Framework to Determine Need for Systemic Chemotherapy in Single-System LCH

The decision for systemic therapy was based on the following factors (Table 1): clinical evidence of active inflammation at the site of lesion (rapid progression, pain and tenderness), functional disability, biochemical evidence of inflammation (ESR, CRP), inflammatory picture on full blood count examination (thrombocytosis, leukocytosis) and the presence of fever. Patients with any CNS-risk lesions received systemic chemotherapy regardless of disease quiescence because the risk of long-term sequelae greatly outweighed any risks associated with systemic chemotherapy.

Figure 3 outlines a clinical decision making framework based on risk organ involvement and disease activity. Given that the patients who required treatment after an initial “wait-and-see” period, progressed within 2 weeks, our proposed duration of the initial “wait-and-see” approach is 4 weeks, during which, patients should be followed up closely to monitor for symptom evolution. Patients who continue to have quiescent disease following this period should then be on follow up until resolution of symptoms albeit less frequently; the average frequency of follow up until resolution in our cohort was 3.1 monthly. Patients should still continue to be monitored for recurrence and other long term sequelae at least 6–12 monthly.

## Systemic Treatment for Active Disease

Patients who received chemotherapy either received Prednisolone and Vinblastine as per the LCH II/III low-risk protocol or oral 6-Mercaptopurine (6-MP) in combination with Methotrexate. Indications for treatment were: (1) thrombocytosis (Platelet count >400 × 10^9^/L) which was the commonest indication for treatment (78.3%), (2) functional disability (30.4%), (3) presence of CNS-risk lesions (26.1%), (4) rapid progression (17.4%), and (5) fever (17.4%).

Specifically, in patients whose indication for treatment included thrombocytosis, we observed a significant decrease in platelet count from a mean of 515.5 × 10^9^ ± SD 88.4 (95% CI 459.3–571.7) at point of diagnosis to 286.7 × 10^9^ ± SD 56 (95% 251.1–322.2) at the end of treatment (*p* < 0.001). Further studies are be required to evaluate if platelet counts correlate with disease activity as decreased platelet count could also be chemotherapy induced.

Although chemotherapy for LCH was generally well-tolerated, 30.4% patients required admissions for neutropenic fever. In addition, 8.7% of patients required port-a-cath insertion in view of difficult venous access for chemotherapy.

## Oral Chemotherapy for Patients With Intermediate Disease Activity

Within the group of patients who received systemic therapy, three patients had received oral chemotherapy with 6-MP and Methotrexate. These patients were deemed to have intermediate disease activity as they had mixed findings of both disease activity and quiescence.

Of these patients, one with multifocal bony lesions had been initially observed with a “wait-and-see” approach but developed tender and rapid progression of some, but not all lesions and thus was started on oral chemotherapy with good response. Another patient had an isolated pelvic bone lesion with functional disability, however as the lesion had decreased in size from time of referral to time of specialist review, with no further biochemical evidence of inflammation, decision was made to start oral chemotherapy instead. The third patient had multifocal bony LCH involving the femur and a depressed skull lesion which strongly suggested near-resolution, however the presence of thrombocytosis and a strong family history of multi-system LCH in an identical twin sibling, prompted the need for systemic therapy.

All three patients treated with oral chemotherapy remained in complete remission without DI, LCH recurrence, or other long-term sequelae.

## Treatment Outcomes: Development of DI and LCH Recurrence

Development of DI was the commonest sequelae on long term follow up (Figure 1). Although none of the patients developed neurodegenerative disease, a patient developed Hodgkin's Lymphoma and another one developed Mollaret's Meningitis, soon after completing therapy for LCH; both remain clinically well on long term follow up having received treatment for their specific conditions.

In the group of patients who received systemic therapy, five had later development of DI after a median duration of 12 months (range: 6–36 months). More than half of these patients had CNS-risk lesions, except one patient with an isolated vertebral lesion who also had leukocytosis, thrombocytosis and a markedly raised ESR (127 mm/h); and one patient with multifocal bony lesions who was initially thought to have quiescent disease but progressed during the “wait-and-see” period and subsequently required systemic chemotherapy.

## Discussion

The results of our analysis suggest that indicators of disease quiescence in SS-LCH can be used to distinguish a subset of patients who may be spared from systemic chemotherapy. The excellent long-term outcomes with a “wait and see” approach demonstrates a benign course with low risk of relapse as compared to those with active SS-LCH requiring treatment and multisystem LCH where there are risks of relapses (17). Further studies are needed to determine the biological differences in this subset of patients with spontaneously resolving SS-LCH.

Based on our albeit limited experience with this “wait-and-see” approach, it appears that patients with active disease would manifest symptoms of progression within a short follow up period of 2 weeks—given that the patients who were initially thought to have quiescent disease progressed within this timeframe. The initial “misclassification” of these four patients also suggests that disease quiescence should not be only evaluated based on isolated findings at a specific timepoint, but rather, after a period of observation for signs of evolution.

The framework to determine disease quiescence was developed based on our institutional clinical experience in treating children with LCH. The main limitation to this study was the small sample size and retrospective cohort study design that limits our ability to statistically validate the scoring system. Nevertheless, it may serve as a framework to guide discerning physicians in deciding on which patients could potentially be spared from chemotherapy. We acknowledge that CNS-risk organ status remains an important factor in determining a patient's risk of long-term sequelae, and that these patients should *not* be managed conservatively regardless of their disease activity as determined by our clinical decision-making framework.

Given that histological diagnosis is the gold standard in LCH, those with presumed LCH were excluded from the main statistical analysis, and only included in the study population if they: (1) did not have any other features to suggest an infective osteolytic lesion or metastatic disease, (2) skeletal survey was performed as part of standard LCH work up and did not show any other evidence of disease involvement elsewhere, (3) that there was sufficient follow up until radiological resolution of the lesion—as resolution would not be expected in cases of other differentials of osteolytic lesions, such as fibrous dysplasia or bone cysts. In half of these patients who did not have a histological diagnosis: the primary lesion was an incidental finding where patients were otherwise asymptomatic. This probably also accounted for the high rate of parental refusal for surgical biopsy and instead, opting for a more conservative “wait-and-see” approach, albeit limiting our analysis. Nevertheless, it is interesting to note the subset of patients who had isolated bony lesions that underwent spontaneous resolution—even without any additional local therapy, such as curettage. Future studies in radiological features of quiescent SS-LCH (18) may reduce need for any surgery in this otherwise benign subtype of LCH.

For patients with “intermediate” disease, the potential efficacy of oral chemotherapy with 6-Mercaptopurine and Methotrexate will be an area of further investigation. Although there is limited data related to long-term toxicity of Prednisolone and Vinblastine, emerging data on the long-term safety of Vincristine, a vinca alkaloid agent similar to Vinblastine, have shown long-term peripheral neuropathy in survivors of acute lymphoblastic leukemia (19).

Overall long-term outcomes in our cohort of patients with single-system LCH is good and mirrors that of other recently published studies (20). In conclusion, this study demonstrates the feasibility and efficacy of a conservative “wait-and-see” approach to a subset of children with quiescent single-system LCH, defined based on clinical features and biochemical indicators of inflammation. This provides a treatment framework to physicians in masterly inactivity in the treatment of LCH.

## Data Availability Statement

The datasets generated for this study will not be made publicly available. Ethics approval of this study does not allow sharing of data.

## Ethics Statement

The studies involving human participants were reviewed and approved by the National Healthcare Group (NHG) Domain Specific Review Board in Singapore.

## Author Contributions

BO, SL, YK, MK, AY, and TQ contributed to the design and implementation of the research, to the analysis of the results, and to the writing of the manuscript. All authors have reviewed and agreed upon the manuscript content.

## Conflict of Interest

The authors declare that the research was conducted in the absence of any commercial or financial relationships that could be construed as a potential conflict of interest.
